# Points in the Arsenical Caustic Treatment of Cutaneous Cancers

**Published:** 1899-03

**Authors:** William S. Gottheil


### Points in the Arsenical Caustic Treatment of Cutaneous Cancers.

BY WILLIAM S. GOTTHEIL, M. D.

1. The arsenious acid caustic treatment of skin cancers does not contemplate or depend upon the actual destruction of the new growth by the caustic.

2. The method is based upon the fact that newly formed tissue of all kinds has less resisting power than the normal structure when exposed to an irritation and its consequent inflammation. Hence the former breaks down under an "insult" which the latter successfully resists.

3. If, therefore, the whole affected area can be subjected to the influence of an irritant of just sufficient strength to cause a reactive inflammation intense enough to destroy the vitality of the new cells, the older normal cells will survive.

4. Arsenious acid of properly mitigated strength is such an agent, and its application causes an inflammation of the required intensity.

5. It therefore exercises a selective influence upon the tissues

*From advance sheets of the third edition of "Jackson on Diseases of the Skin." Lea Brothers & Co., publishers.

to which it is applied, and causes the death of the cancer cells in localities outside the apparent limits of the new growth, where there is as yet no evidence of disease.

6. It is superior, in suitable cases, to any method, knife or cautery, which requires the exercise of the surgeon's judgment as to the extent to which it is to be carried. That that judgment is often wrong, and necessarily so, is shown by the frequency of recurrence under these methods even in the best hands.

7. It is applicable to all cutaneous carcinomata in which the deeper structures are not involved, and which do not extend far onto the mucous membranes.

8. It is easy of application; it is safe; it is only moderately painful; and its results compare favorably with those obtained with other methods.

